# Retained Intrauterine Fetal Bone Fragments Causing Secondary Infertility: A Review

**DOI:** 10.7759/cureus.44005

**Published:** 2023-08-23

**Authors:** Lucky Srivani Reddy, Arpita Jaiswal, Kavyanjali Reddy, Garapati Jyotsna, Pallavi Yadav

**Affiliations:** 1 Obstetrics and Gynecology, Jawaharlal Nehru Medical College, Datta Meghe Institute of Higher Education and Research, Wardha, IND; 2 Surgery, Jawaharlal Nehru Medical College, Datta Meghe Institute of Higher Education and Research, Wardha, IND

**Keywords:** pregnancy outcomes, endometrial receptivity, inflammation, diagnostic challenges, hysteroscopy, fertility, secondary infertility, retained intrauterine fetal bone fragments

## Abstract

Retained intrauterine fetal bone fragments are emerging as a potential yet often overlooked cause of secondary infertility, posing significant challenges for couples who have previously experienced successful pregnancies. This review article explores the association between retained fetal bone fragments and secondary infertility by delving into their impact on fertility, pregnancy outcomes, and diagnostic challenges. The review highlights the underlying mechanisms of fragment retention, including immune response and inflammation, and their detrimental effects on endometrial receptivity and implantation. The diagnostic difficulties and importance of specialized imaging techniques like hysteroscopy for accurate diagnosis are also discussed. The article also provides insights into available treatment options, such as medical management and surgical interventions, focusing on hysteroscopy as the gold standard for diagnosis and treatment. The implications for clinical practice emphasize early diagnosis and intervention to improve fertility outcomes and reduce the emotional burden of secondary infertility. Furthermore, the review discusses preventive strategies and the potential for future research to refine diagnostic methods and explore novel treatments. By recognizing and addressing the impact of retained fetal bone fragments, this review aims to enhance the understanding and management of this condition, providing valuable support to couples seeking to overcome the challenges of secondary infertility on their journey toward parenthood.

## Introduction and background

Secondary infertility refers to the inability of a couple to conceive or carry a pregnancy to term after having one or more successful pregnancies in the past. Unlike primary infertility, where a couple has never achieved a pregnancy, secondary infertility poses unique challenges and emotional burdens. It affects millions of couples worldwide and can have various underlying causes [1-4]. Retained intrauterine fetal bone fragments occur when small pieces of fetal bone remain in the uterus following a previous pregnancy loss, such as a miscarriage or an incomplete abortion. These fragments can result from a spontaneous miscarriage in which not all products of conception are expelled, or they may persist after surgical procedures like dilation and curettage (D&C) [5-7]. 

The presence of retained fetal bone fragments in the uterus can significantly impact a woman's fertility. These fragments may cause persistent inflammation and scarring within the uterine cavity, disrupting the delicate balance necessary for successful implantation and pregnancy. Consequently, couples affected by this condition may experience difficulty achieving a subsequent pregnancy, leading to secondary infertility [8]. Moreover, retained fetal bone fragments can also contribute to recurrent miscarriages, as they interfere with the normal development of a healthy pregnancy. Understanding the implications of this condition is crucial for healthcare providers to offer timely diagnosis and appropriate management to affected couples. 

This review aims to comprehensively examine the link between retained intrauterine fetal bone fragments and secondary infertility. Secondary infertility, a distressing condition faced by couples who have previously experienced successful pregnancies, can be attributed to various underlying factors. This review will delve into the definition and explanation of retained fetal bone fragments, exploring how they can arise from incomplete abortions or other procedures. Understanding the importance and impact of these fragments on fertility is crucial as they may lead to inflammation, scarring, and hinder implantation. By elucidating the causes, diagnostic challenges, and management options, we aim to provide a comprehensive resource for healthcare professionals and researchers. Ultimately, this review aims to contribute to the advancement of knowledge in this area and foster improved diagnostic and therapeutic approaches for couples facing the challenges of retained intrauterine fetal bone fragments and their implications on their journey toward parenthood. 

## Review

Causes and risk factors of retained intrauterine fetal bone fragments 

Incomplete Abortion and Missed Miscarriage 

Incomplete abortion and missed miscarriage are common occurrences in early pregnancy. In an incomplete abortion, the pregnancy terminates, but not all products of conception, including fetal tissues like bones, are fully expelled from the uterus. This can happen spontaneously or due to a medical or surgical abortion procedure that is not entirely successful in removing all the fetal remnants [9]. 

Similarly, a missed miscarriage refers to the death of the fetus within the uterus, but the body does not recognize the loss immediately. As a result, the process of miscarriage may not occur naturally, leaving fetal tissues behind [10]. In both cases, retained intrauterine fetal bone fragments can persist within the uterine cavity after the pregnancy loss. These fragments are not spontaneously expelled, as they should be during a normal miscarriage or abortion process. The presence of these retained fragments can have several implications for future fertility [11]. 

First, the fragments can cause chronic inflammation within the uterine cavity. The immune system recognizes the retained fetal tissues as foreign, leading to an inflammatory response. This inflammation can disrupt the normal endometrial environment, making it less receptive to embryo implantation. 

Second, the presence of retained fetal bone fragments can lead to scarring or adhesions within the uterine cavity. This scarring can alter the uterine structure and may interfere with the implantation of a fertilized embryo. Scarring may also affect the endometrium's blood supply, compromising its ability to support a developing pregnancy [12]. Additionally, the retained fragments can create a hostile environment for the embryo. They may physically obstruct the embryo's attachment to the endometrial lining, reducing the chances of successful implantation. Furthermore, the fragments may release substances that are harmful to embryo development, increasing the risk of early pregnancy loss or recurrent miscarriages [13]. 

Dilation and Curettage (D&C) Procedures 

D&C is a widely used surgical procedure for various purposes in women's healthcare. One of its primary applications is to remove any remaining fetal tissue after a miscarriage or pregnancy termination, providing both physical and emotional closure to the patient. Additionally, D&C can be employed for diagnostic purposes, such as investigating abnormal uterine bleeding or detecting and treating certain gynecological conditions [14]. 

Despite being generally considered safe and routine, D&C does carry some potential risks. One of the major concerns is the possibility of retained fetal bone fragments if the procedure is not performed thoroughly or if small fragments are inadvertently left behind in the uterus. These retained fragments can lead to complications, including infection, persistent bleeding, or discomfort for the patient. Therefore, the healthcare provider must exercise utmost care and precision during the D&C procedure to minimize the risk of such complications [15]. 

To address this issue, medical practitioners must be skilled and experienced in performing D&C procedures, ensuring the complete removal of all fetal tissue and bone fragments. Furthermore, post-procedure monitoring and follow-up care are crucial to identify any signs of potential complications and to take timely action if necessary. Patient education about the procedure and its possible outcomes can also help manage expectations and promote cooperation in postoperative care, ultimately contributing to a successful recovery and improved patient well-being [16]. 

Other Potential Risk Factors Associated With Retained Fetal Bone Fragments 

In addition to incomplete abortion and D&C procedures, several other potential risk factors can lead to the retention of fetal bone fragments in the uterus. One significant factor is uterine abnormalities, which can disrupt the normal process of uterine contraction and expulsion of fetal tissue. Such abnormalities might include a bicornuate or septate uterus, which can create spaces or partitions within the uterus, making it difficult for the uterine walls to contract effectively. 

Another risk factor is the presence of intrauterine adhesions, also known as Asherman's syndrome, which is characterized by the formation of scar tissue inside the uterus. These adhesions can develop due to previous uterine surgeries, infections, or trauma and can obstruct the natural expulsion of fetal remains during a miscarriage [17]. 

Anatomical variations can also hinder the complete expulsion of fetal tissues after a pregnancy loss. Some women may have anatomical differences in their cervix or uterus that prevent the smooth passage of fetal debris during miscarriage [18]. 

Furthermore, certain medical conditions such as chronic endometritis (inflammation of the uterine lining), gestational trophoblastic disease (abnormal growth of placental tissue), or clotting disorders can interfere with the body's ability to expel fetal bone fragments completely [19]. Identifying and understanding these additional risk factors is crucial for healthcare providers when managing patients who experience retained fetal bone fragments. Proper diagnosis and appropriate medical interventions are essential to prevent potential complications, such as infection, prolonged bleeding, or fertility issues, and to ensure the affected individual's overall reproductive health and well-being [6]. 

Impact of Retained Fetal Bone Fragments on the Uterus and Fertility 

The presence of retained intrauterine fetal bone fragments can profoundly impact the uterus and fertility. These fragments may trigger persistent inflammation and cause scarring within the uterine cavity, disrupting the normal endometrial environment essential for successful implantation and pregnancy maintenance. Additionally, the inflammatory response to the retained fragments can compromise the uterine lining's receptivity to an embryo, further reducing the chances of conception [8]. 

Moreover, retained fetal bone fragments can contribute to recurrent miscarriages, as they interfere with the normal development of a healthy pregnancy. The prolonged presence of these fragments in the uterus can lead to chronic endometritis, adversely affecting the implantation process and increasing the risk of implantation failure [20]. Understanding the causes and risk factors of retained intrauterine fetal bone fragments and their impact on the uterus and fertility is crucial in effectively diagnosing and managing this condition. Addressing these aspects can aid healthcare providers in offering appropriate interventions and personalized treatment options to couples experiencing secondary infertility due to this challenging reproductive issue [15]. 

Clinical presentation and diagnosis 

Symptoms and Signs of Retained Fetal Bone Fragments 

Retained intrauterine fetal bone fragments refer to fragments of fetal bones that remain within the uterus after a previous pregnancy or miscarriage. The clinical presentation of this condition can vary, and while some individuals may not experience any noticeable symptoms, others may manifest specific signs indicating its presence [21]. 

One of the primary symptoms associated with retained intrauterine fetal bone fragments is abnormal vaginal bleeding. Women may experience irregular or prolonged bleeding, often characterized by spotting or heavy menstrual-like bleeding. This occurs because the presence of the fragments can disrupt the normal uterine lining, leading to irregular shedding and bleeding patterns [22]. Additionally, individuals with this condition may suffer from pelvic pain or discomfort. The presence of retained fragments can cause inflammation or irritation within the uterus, leading to persistent pelvic pain, cramping, or discomfort. 

In some cases, retained fetal bone fragments may be a hidden cause of unexplained infertility. Couples trying to conceive may face difficulty in achieving pregnancy, despite no apparent reasons for their fertility struggles. The fragments can interfere with implantation, disrupt the uterine environment, and impede successful conception [5]. Furthermore, retained intrauterine fetal bone fragments can contribute to recurrent miscarriages. The presence of these fragments can disrupt the normal development of a subsequent pregnancy, leading to multiple miscarriages and causing significant emotional distress to the affected individuals. 

It's important to note that while some individuals may remain asymptomatic, others may experience one or more of these symptoms. If a woman experiences any of the mentioned signs or suspects the presence of retained fetal bone fragments, seeking medical attention and proper evaluation by a healthcare professional is essential. Treatment options can include medications, minimally invasive procedures, or surgery, depending on the severity of the condition and the individual's specific circumstances. Early diagnosis and appropriate management can help improve the chances of a successful pregnancy outcome and alleviate associated symptoms. 

Diagnostic Challenges and Difficulties in Diagnosing the Condition 

Diagnosing retained intrauterine fetal bone fragments can be challenging due to several factors. First, some individuals may not exhibit any symptoms, creating a significant hurdle in identifying the presence of these fragments without appropriate diagnostic tests. Asymptomatic cases can easily go unnoticed, delaying diagnosis and potentially causing complications if left untreated [7]. 

Second, the symptoms of retained fetal bone fragments can overlap with those of other gynecological conditions, such as endometriosis or uterine fibroids. These similarities may lead to misdiagnosis or delayed diagnosis, as healthcare providers might focus on more common conditions rather than considering retained fragments as a potential cause [23]. 

Thirdly, there might be a lack of awareness among healthcare providers about the possibility of retained fetal bone fragments causing secondary infertility. This lack of consideration could result in underdiagnosis, with patients remaining unaware of the true underlying cause of their fertility issues [24]. Finally, the available diagnostic tools might not always be adequate in detecting small fragments or providing enough information for accurate diagnosis. Standard imaging techniques may fail to identify tiny bone fragments, leading to a false negative diagnosis or inconclusive results. 

In light of these challenges, it is crucial to promote awareness among healthcare professionals about the possibility of retained intrauterine fetal bone fragments and to develop more advanced and sensitive diagnostic methods to improve early detection and appropriate management of this condition. Early diagnosis can lead to timely intervention and better outcomes for affected individuals seeking fertility treatments or relief from related symptoms. 

Imaging Techniques and Diagnostic Tools Used in Diagnosis 

Diagnosing retained intrauterine fetal bone fragments requires a comprehensive approach, and healthcare providers utilize imaging techniques and diagnostic tools to ensure accuracy. One such technique is transvaginal ultrasound (TVUS), a non-invasive imaging method that provides a clear visualization of the uterus and its contents. Through TVUS, healthcare providers can identify and assess the size and location of the retained fetal bone fragments [25]. 

Another valuable tool in diagnosing this condition is hysteroscopy, where a thin, lighted tube called a hysteroscope is inserted into the uterus to visualize its interior directly. This procedure allows for identifying the retained fragments and enables their removal during the same intervention, contributing to a more effective diagnosis and treatment process [26]. 

Hysterosalpingography (HSG) is another radiologic procedure used to diagnose this condition. In HSG, a contrast dye is injected into the uterine cavity and fallopian tubes, helping healthcare providers identify any abnormalities, including retained fetal bone fragments. Additionally, it assists in evaluating the shape and size of the uterine cavity, providing valuable information for diagnosis and treatment planning [27]. 

In more complex cases or when other diagnostic methods are inconclusive, magnetic resonance imaging (MRI) may be employed. MRI can offer additional information on the uterine structure, aiding in accurately diagnosing retained fetal bone fragments [28]. Accurate diagnosis of this condition is crucial for developing effective treatment plans and improving fertility outcomes for those affected. To achieve this, healthcare providers adopt a multidisciplinary approach that combines clinical history, physical examination, and the appropriate use of diagnostic tools. This comprehensive approach enhances the accuracy of the diagnosis. It allows for implementing tailored management strategies, increasing the likelihood of successful outcomes for patients dealing with retained intrauterine fetal bone fragments [29]. 

Pathophysiology 

Mechanisms of Fetal Bone Fragment Retention 

Retaining intrauterine fetal bone fragments involves various mechanisms, often associated with incomplete pregnancy loss or surgical procedures. After a miscarriage or incomplete abortion, fetal tissue, including bone fragments, may not be fully expelled from the uterine cavity. The reasons for retention can include ineffective uterine contractions, anatomical variations, or adhesions within the uterine cavity, which prevent the complete evacuation of fetal remnants. Additionally, during D&C procedures, small bone fragments might be inadvertently left behind due to technical challenges or inadequate examination [5]. 

Immune Response and Inflammation 

Retained fetal bone fragments can trigger an inflammatory response within the uterine cavity. The immune system recognizes the retained foreign tissue as antigens, leading to the recruitment of immune cells and the release of inflammatory mediators. Chronic inflammation within the endometrial lining can adversely affect endometrial receptivity, compromising the normal processes required for successful embryo implantation. The inflammatory environment may also contribute to the formation of intrauterine adhesions (synechiae) or scarring, further hindering fertility [30]. 

Effect on Endometrial Receptivity and Implantation 

The presence of retained fetal bone fragments disrupts the receptive environment of the endometrial lining. The endometrium undergoes dynamic changes during the menstrual cycle, preparing for embryo implantation. However, chronic inflammation and altered cytokine levels due to retained fragments can affect the normal development of the endometrial lining. This altered endometrial environment may impair embryo implantation, leading to implantation failure and secondary infertility [31]. 

Moreover, the physical presence of fetal bone fragments within the uterine cavity can create a hostile environment for embryo development. The fragments may interfere with the embryo's ability to attach to the endometrial lining, reducing the chances of successful implantation and subsequent pregnancy. The pathophysiology of retained intrauterine fetal bone fragments and their impact on immune responses, inflammation, and endometrial receptivity is complex and multifaceted. Understanding these mechanisms is crucial for tailoring effective therapeutic approaches to address the underlying causes of secondary infertility associated with this condition. Healthcare providers can offer personalized treatment strategies to improve fertility outcomes for couples affected by retained fetal bone fragments by targeting the pathophysiological processes involved [32]. 

Association with secondary infertility 

Understanding Secondary Infertility and Its Prevalence 

Secondary infertility is when a couple experiences difficulty conceiving or carrying a pregnancy to term after having one or more successful pregnancies. It is essential to differentiate secondary infertility from primary infertility, where a couple has never achieved a pregnancy. Secondary infertility can be emotionally distressing for couples who had previously experienced the joy of parenthood and now struggle to expand their family [33]. 

The prevalence of secondary infertility varies among populations and is influenced by factors such as maternal age, reproductive health, and cultural norms regarding family size. Studies have reported that the prevalence of secondary infertility is comparable to or even higher than primary infertility rates, making it a significant reproductive health concern [34]. 

*Link Between Retained Fetal Bone Fragments and Secondary Infertility* 

Retained intrauterine fetal bone fragments have emerged as a potential yet often overlooked cause of secondary infertility. The presence of these fragments within the uterine cavity can disrupt the normal physiological processes required for successful conception and pregnancy. While not all cases of secondary infertility can be attributed to retained fetal bone fragments, it is crucial to consider this condition in the diagnostic workup of affected couples [6]. 

Impact on Fertility and Pregnancy Outcomes 

The association between retained fetal bone fragments and secondary infertility is primarily attributed to these fragments' detrimental effects on the endometrial receptivity and implantation process. The chronic inflammation and scarring caused by the presence of fetal bone fragments create an unfavorable environment within the uterine cavity, making it challenging for embryos to implant and develop normally [35]. 

Furthermore, the inflammatory response elicited by retained fragments can disrupt the balance of hormones and growth factors necessary for folliculogenesis, ovulation, and endometrial preparation. This disruption may lead to ovulatory dysfunction and irregular menstrual cycles, further complicating conception [36]. In cases where conception occurs despite the presence of retained fetal bone fragments, the risk of adverse pregnancy outcomes, such as recurrent miscarriages, is heightened. The inflammatory milieu within the uterus may adversely affect embryo development and placental function, increasing the likelihood of pregnancy loss [37]. 

Management and treatment 

Strategies for Diagnosing Retained Fetal Bone Fragments 

Accurate and timely diagnosis is crucial for effectively managing retained intrauterine fetal bone fragments and addressing associated secondary infertility. When faced with a patient exhibiting symptoms suggestive of this condition, healthcare providers employ a series of strategies to ensure a precise diagnosis [24]. 

First, a thorough medical history focuses on the patient's obstetric and gynecological background. This comprehensive review can provide valuable insights into any past pregnancies and miscarriages, which may be relevant to the current presentation [38]. 

Next, a physical examination, particularly a pelvic examination, is performed to check for any signs of inflammation or uterine abnormalities. Although this examination may not be sufficient for a definitive diagnosis, it can provide additional clues [39]. 

TVUS is often the initial imaging modality used to assess the uterine cavity. This non-invasive technique allows healthcare providers to identify retained fetal bone fragments' presence, location, and size. TVUS plays a crucial role in the diagnostic process [40]. 

For a more definitive diagnosis and therapeutic intervention, hysteroscopy is considered the gold standard. Hysteroscopy involves the insertion of a thin, lighted tube through the vagina and cervix into the uterus, allowing direct visualization of the uterine cavity. During the same procedure, retained fragments can be removed, thereby offering both diagnosis and treatment [41]. In some cases, HSG may also be utilized to evaluate the uterine cavity. However, it may not be as sensitive as hysteroscopy in identifying small bone fragments, making it a less preferred option. 

Medical and Surgical Management Options 

Managing retained intrauterine fetal bone fragments causing secondary infertility requires careful consideration of various factors. These factors include the size and location of the fragments, the extent of inflammation or scarring in the uterus, and the couple's reproductive goals. Two main treatment options are available [5]. 

A conservative approach known as medical management may be chosen for cases where the fragments are small and asymptomatic. This approach involves closely monitoring the patient's condition and administering anti-inflammatory medications to reduce inflammation and alleviate symptoms. While medical management can be effective in some instances, it may not always resolve the underlying cause of secondary infertility, particularly if the bone fragments significantly impact the endometrial receptivity required for successful conception [42]. 

On the other hand, for larger or symptomatic retained fetal bone fragments, surgical intervention, specifically hysteroscopy, is the preferred course of action. Hysteroscopy is a minimally invasive procedure that allows direct visualization of the uterus using a thin, lighted instrument called a hysteroscope. Through this procedure, the surgeon can locate and remove the fragments, improving the endometrial environment and increasing the chances of conception. Hysteroscopy is generally well-tolerated by patients and associated with minimal risks [43]. 

Ultimately, the choice between medical management and surgical intervention depends on the individual circumstances of each case. In consultation with the couple, the medical team will carefully assess the specific situation and recommend the most appropriate treatment approach to address the retained fetal bone fragments and help achieve the couple's reproductive goals. Early detection and intervention are crucial in optimizing the chances of successful conception and restoring fertility in cases of secondary infertility caused by retained intrauterine fetal bone fragments [44]. 

Treatment Outcomes and Success Rates 

The success of treatment for retained intrauterine fetal bone fragments depends on various factors, including the extent of inflammation or scarring, the effectiveness of the chosen treatment method, and the individual patient's response [8]. In cases where hysteroscopy is performed to remove retained fragments, studies have shown favorable outcomes, with many patients experiencing improved fertility and successful pregnancies following the procedure. Restoring a healthier uterine environment after fragment removal enhances the chances of successful embryo implantation and pregnancy maintenance [45]. 

However, it is essential to note that not all cases of secondary infertility associated with retained fetal bone fragments may be fully resolved with treatment. Some individuals may continue to face challenges in conceiving, especially if other underlying fertility factors are at play. Further fertility evaluations and personalized treatment plans may be necessary [6]. 

Prevention and prognosis 

Strategies to Reduce the Risk of Retained Fetal Bone Fragments 

While not all cases of retained intrauterine fetal bone fragments can be prevented, certain strategies can help reduce the risk of this condition and its impact on fertility. The first crucial step is ensuring comprehensive pregnancy care, where pregnant individuals receive adequate prenatal monitoring. Early detection and management of complications can help minimize the risk of incomplete abortions, thereby reducing the likelihood of retained fetal bone fragments [7]. 

Minimizing the need for D&C is another essential approach. Healthcare providers should opt for conservative management approaches, such as expectant or medical management, to avoid unnecessary D&C procedures that may contribute to the retention of bone fragments [14]. 

Furthermore, improved surgical techniques play a pivotal role in preventing the issue. Whenever D&C or other surgical interventions are necessary, healthcare providers should use meticulous and thorough techniques to minimize the chances of leaving behind fragments [46]. Incorporating enhanced imaging techniques is also beneficial. Utilizing advanced imaging modalities like 3D ultrasound or MRI can aid in identifying and managing retained fetal bone fragments with greater precision, improving diagnosis and treatment outcomes [47]. 

Hysteroscopy-guided interventions offer a comprehensive solution. Hysteroscopy not only enables the diagnosis of retained fetal bone fragments but also allows for their simultaneous treatment. Therefore, hysteroscopic procedures should be considered a first-line approach whenever feasible, as they can effectively diagnose and resolve the issue [48]. By combining these strategies, healthcare providers can significantly reduce the occurrence of retained intrauterine fetal bone fragments and potentially mitigate their impact on fertility, promoting safer and healthier pregnancies. However, as with any medical condition, individual circumstances may vary, and prompt and appropriate medical attention should always be sought when needed [5]. 

The prognosis for Fertility After Successful Treatment 

The prognosis for fertility after successful treatment of retained intrauterine fetal bone fragments can be favorable for many couples. Removing the retained fragments through hysteroscopy or other surgical interventions improves the uterine environment, reducing inflammation and enhancing endometrial receptivity [5]. After successful treatment, couples may experience improved fertility, increased chances of embryo implantation, and reduced risk of recurrent miscarriages. Many individuals have achieved successful pregnancies and delivered healthy babies after appropriately managing this condition [49]. 

However, it is essential to remember that fertility treatment success depends on various factors, including any other underlying fertility issues or reproductive health concerns. In some cases, other infertility factors may coexist, necessitating additional treatment or assisted reproductive technologies (ART) to achieve pregnancy [50]. 

Moreover, the individual response to treatment can vary, and not all couples may achieve pregnancy immediately after addressing retained fetal bone fragments. Patience, ongoing monitoring, and personalized fertility support may be required for those still facing challenges in conceiving even after successful treatment [51]. 

Diagnostic challenges and management 

Difficulties in Diagnosing Retained Fetal Bone Fragments 

Diagnosing retained intrauterine fetal bone fragments can be challenging due to various factors complicating the identification and understanding of this condition. One significant hurdle lies in the existence of asymptomatic cases, where affected individuals may not experience noticeable symptoms. As a result, healthcare providers may not consider this condition during routine evaluations, leading to underdiagnosis [24]. 

Adding to the complexity are the overlapping symptoms with other gynecological conditions, such as uterine fibroids or endometriosis. The similarities in symptoms can cause confusion and may result in misdiagnosis or delayed diagnosis, potentially prolonging the patient's suffering and preventing timely intervention [52]. 

Another contributing factor to the diagnostic challenge is the lack of awareness among healthcare providers regarding the association of retained fetal bone fragments with secondary infertility. Failing to consider this condition while evaluating infertility cases can lead to overlooking the presence of retained fragments as a possible contributing factor [53]. 

Furthermore, conventional imaging techniques, like TVUS, might not always be sensitive enough to detect small bone fragments within the uterus. This limitation necessitates using more specialized imaging methods or direct visualization through hysteroscopy, which may not be routinely employed in all healthcare settings [54]. 

*Evaluation and Workup for Secondary Infertility* 

The evaluation and workup for secondary infertility are crucial steps to identify potential underlying causes, including rare but significant factors such as retained intrauterine fetal bone fragments. Secondary infertility is the inability to conceive or carry a pregnancy to term after having at least one successful pregnancy. When faced with such challenges, the following steps are generally involved in the diagnostic process to determine the cause; first, obtaining a comprehensive medical history is paramount. This involves gathering detailed information about the patient's previous pregnancies, miscarriages, and any fertility-related issues they might have experienced. Understanding the patient's reproductive health in the context of their past pregnancies can provide valuable insights into their current fertility concerns [6]. 

Second, a physical examination, specifically a pelvic examination, is conducted. This helps healthcare providers detect any signs of inflammation, adhesions, or other abnormalities in the reproductive organs that could indicate the presence of retained fetal bone fragments or other potential causes of secondary infertility [5]. Hormonal and ovulatory assessment is the next step. This involves evaluating the patient's hormone levels and ovulation patterns to identify any hormonal imbalances or ovulatory dysfunction that might contribute to their infertility [55]. 

TVUS is often the initial imaging technique used in the evaluation process. It helps assess the uterine cavity for any retained fetal bone fragments or other abnormalities that may be affecting fertility [56]. In cases where TVUS findings are inconclusive or when there is a high suspicion of retained fragments, hysteroscopy is recommended. Hysteroscopy is a minimally invasive procedure in which a thin, lighted tube with a camera is inserted into the uterus through the vagina and cervix. It allows for direct visualization and removal of retained fetal bone fragments, thus simultaneously enabling diagnosis and treatment [57]. 

Treatment Options for Retained Fetal Bone Fragments 

Treatment options for retained intrauterine fetal bone fragments depend on various factors, including the size and location of the fragments, the extent of inflammation or scarring, and the patient's reproductive goals. The two main approaches to managing this condition are medical management and surgical interventions [5]. For asymptomatic or small fragments, medical management might be a viable option. This approach involves close monitoring of the condition and using anti-inflammatory medications to reduce inflammation and manage any associated symptoms. However, it is important to note that medical management alone may not be sufficient in cases where significant inflammation or larger retained fragments are present, especially when secondary infertility is a concern [58]. 

In cases where medical management is not practical or when there are larger retained fragments causing issues, surgical interventions become necessary. Among these, hysteroscopy stands out as the gold standard both for diagnosing and treating the condition. During a hysteroscopy, the fragments can be directly visualized and removed, leading to an improved uterine environment, which enhances the chances of successful embryo implantation and subsequent pregnancy [59]. 

Other surgical procedures like D&C or hysteroscopic-guided evacuation may be considered in specific cases, depending on the individual circumstances of the patient. The decision to opt for medical management or surgical intervention is influenced by various factors, including the patient's clinical presentation, fertility goals, and the healthcare provider's expertise. Hysteroscopy-guided interventions offer the advantage of diagnosing and treating the condition in a single procedure, reducing the need for additional interventions and potentially enhancing the overall outcome. Ultimately, the choice of treatment approach should be made collaboratively between the patient and their healthcare team to ensure the best possible outcome for the patient's reproductive health [60]. 

## Conclusions

In conclusion, this review article has highlighted the significant association between retained intrauterine fetal bone fragments and secondary infertility. Secondary infertility, a distressing condition faced by couples after experiencing successful pregnancies, can be attributed to various underlying factors. Among them, retained fetal bone fragments emerge as a potential yet often overlooked cause, impacting fertility and pregnancy outcomes. The review outlined the mechanisms of fragment retention, the immune response and inflammation triggered by their presence, and their adverse effects on endometrial receptivity and implantation. We discussed the diagnostic challenges associated with asymptomatic cases and overlapping symptoms, emphasizing the importance of specialized imaging techniques such as hysteroscopy for accurate diagnosis. Moreover, the review explored the available treatment options, including medical management and surgical interventions like hysteroscopy, which have shown favorable outcomes in improving fertility for affected couples. It is evident that early diagnosis and intervention play a crucial role in enhancing fertility outcomes and reducing the emotional burden of secondary infertility. The implications for clinical practice underscore the need for healthcare providers to consider retained fetal bone fragments during fertility evaluations and to adopt preventive strategies to minimize the risk of this condition. Future research should continue to refine diagnostic methods and explore novel treatments, fostering advancements in the management of this condition and offering hope to couples seeking to build their families. Ultimately, by recognizing and addressing the impact of retained fetal bone fragments, we can strive to provide better care and support for those navigating the challenges of secondary infertility on their path to parenthood.
